# Microfabricated intracortical extracellular matrix-microelectrodes for improving neural interfaces

**DOI:** 10.1038/s41378-018-0030-5

**Published:** 2018-09-24

**Authors:** Wen Shen, Suradip Das, Flavia Vitale, Andrew Richardson, Akshay Ananthakrishnan, Laura A. Struzyna, Daniel P. Brown, Naixin Song, Murari Ramkumar, Timothy Lucas, D. Kacy Cullen, Brian Litt, Mark G. Allen

**Affiliations:** 1 Krishna P. Singh Center for Nanotechnology, University of Pennsylvania, Philadelphia, PA 19104 USA; 2 Department of Neurosurgery, Perelman School of Medicine, University of Pennsylvania, Philadelphia, PA 19104 USA; 3 Department of Neurology, Perelman School of Medicine, University of Pennsylvania, Philadelphia, PA 19104 USA; 4 Department of Mechanical Engineering and Applied Mechanics, School of Engineering and Applied Science, University of Pennsylvania, Philadelphia, PA 19104 USA; 5 Department of Bioengineering, School of Engineering and Applied Science, University of Pennsylvania, Philadelphia, PA 19104 USA; 6 Department of Electrical and Systems Engineering, School of Engineering and Applied Science, University of Pennsylvania, Philadelphia, PA 19104 USA; 7 Department of Materials Science and Engineering, School of Engineering and Applied Science, University of Pennsylvania, Philadelphia, PA 19104 USA; 8 Present Address: Department of Mechanical and Aerospace Engineering, University of Texas at Arlington, Arlington, TX 76019 USA

## Abstract

Intracortical neural microelectrodes, which can directly interface with local neural microcircuits with high spatial and temporal resolution, are critical for neuroscience research, emerging clinical applications, and brain computer interfaces (BCI). However, clinical applications of these devices remain limited mostly by their inability to mitigate inflammatory reactions and support dense neuronal survival at their interfaces. Herein we report the development of microelectrodes primarily composed of extracellular matrix (ECM) proteins, which act as a bio-compatible and an electrochemical interface between the microelectrodes and physiological solution. These ECM-microelectrodes are batch fabricated using a novel combination of micro-transfer-molding and excimer laser micromachining to exhibit final dimensions comparable to those of commercial silicon-based microelectrodes. These are further integrated with a removable insertion stent which aids in intracortical implantation. Results from electrochemical models and in vivo recordings from the rat’s cortex indicate that ECM encapsulations have no significant effect on the electrochemical impedance characteristics of ECM-microelectrodes at neurologically relevant frequencies. ECM-microelectrodes are found to support a dense layer of neuronal somata and neurites on the electrode surface with high neuronal viability and exhibited markedly diminished neuroinflammation and glial scarring in early chronic experiments in rats.

## Neural electrodes: biocompatible interfacing

Neural microelectrodes composed of extracellular matrix proteins enable biocompatible interfaces that reduce inflammatory reactions, and allow dense neuronal surface activity. Neuroscience research relies upon direct interfacing of electrodes with neural microcircuits. However, inflammatory response in the host organism is a major challenge, as is ensuring complete interfacing with the electrode. A team from University of Pennsylvania led by Mark Allen now use extracellular matrix proteins at the interface between the electrode and the organism, fabricated via micro-molding and transfer, and micro-machining. In vivo studies of the cortex region of a rat suggest that protein encapsulation of the electrode has no negative effect on impedance measurements, and can support a dense layer of neuronal somata and neutrites on the electrode surface. Diminished neuroinflammation suggests the possibility to apply these electrodes in clinical situations.

## Introduction

The capability of intracortical neural microelectrodes to obtain action potentials from local neural microcircuits makes them an indispensable tool for accurate clinical diagnoses and treatment of neurological disorders, neuroscientific electrophysiological research, and applications such as brain computer interfaces (BCI)^1,2^. Silicon or metal-based intracortical neural microelectrodes have demonstrated significant successes in neuronal recording^3^, deep brain stimulation^4^, and neuronally controlled prosthetics to restore lost sensory and motor abilities such as robotic arm movement^5–7^, visual function^8^, speech function^9^, etc. However, these inorganic devices also have several shortcomings, including poor biocompatibility and large mechanical mismatch with host neural tissue, which can lead to damage of local neuronal environments, inflammation, and scar formation^10–13^. These issues have driven the investigation of new materials for microelectrodes with emphasis on greater neural compatibility and mechanical flexibility.

It is possible to achieve these complementary requirements by building interfaces out of protein-based composite materials^14^. Type I collagen (collagen I, for short), due to fibrillary structures, provides mechanical support to resident cells in the extracellular matrix (ECM)^15,16^, It has already been used in many applications such as wound healing^17^ and soft tissue repair^18^; however, its application in the brain alone as neural interfaces has not been demonstrated. Among the major ECM proteins in the brain tissue, fibronectin assists the cell–matrix adhesions through specific peptide sequences^19,20^, laminin and Type IV collagen (collagen IV, for short) not only form two-dimensional networks in basal laminae, but also interact with many cell surface proteins and regulate development, differentiation, and cell migration^20,21.^

Previously, nanoscale coatings using ECM components such as laminin and fibrin on the surface of silicon- or metal-based neural electrodes have been realized through passive adsorption^22^ and covalent immobilization^23^. These ECM-coated electrodes were reported to have reduced inflammation and supported survival of viable neurons^22,23^, However, these effects only persisted for approximately 1 week, after which the ECM coatings were observed to be dissolved^22,23^, This indicates that merely coating ECM materials onto microelectrodes may be insufficient in mitigating the evolution of the inflammation processes at neural interfaces^24^. Instead, developing microelectrodes which are primarily composed of ECM materials may facilitate sustained stability and biocompatibility of microelectrode interfaces.

Although ECM-mimics have been fabricated using micro-patterning approaches including coating, molding, electrospinning, and wet-spinning^25^, the production of stand-alone, natural materials-based microelectronic devices with sizes in the range of tens to hundreds of micrometers (in the size range of neurons) remains to be achieved^25–27^. To address this apparent gap, we have developed ECM composites that are predominantly comprised of collagen I and functional components of the brain tissue, such as, laminin, fibronectin, and collagen IV. As is the case with most natural tissues, the fibrillar structure of collagen I plays a key role in providing structural support to the ECM-encapsulated microelectrodes developed in this study, while the functional components provide biological cues to regulate cellular adhesion and potentially further depress inflammatory responses at the neural interfaces. We have developed a batch microfabrication technology, which can enable these thick ECM composites to effective encapsulate the recording components of ECM-microelectrodes and facilitate their integration with removable insertion stents. Using this rapid fabrication approach, ECM-microelectrodes with sizes comparable to commercially available Si-microelectrodes (Michigan M15, NeuroNexus Technologies, Inc., Ann Arbor, MI, USA) have been produced. Their biocompatibility and interface stability have been comprehensively evaluated in vitro for neural culture viability, neuronal adhesion, growth, and network formation. Unlike laminin, fibronectin, and collagen IV, collagen I is not a natural constituent of the ECM found in brain tissues. Therefore, the local host cell/tissue responses to ECM-microelectrodes composed of collagen I (collagen I microelectrodes) post implantation have been evaluated. In addition, the influences of ECM materials on the electrochemical performance of the ECM-microelectrodes are studied using electrochemical impedance spectroscopy (EIS) and described using new circuit models. Their neural recording capability has been demonstrated in vivo in the rat cortex region.

## Results

### Microfabricated ECM-microelectrodes

Micro-transfer-molding has been demonstrated as an appropriate bottom-up approach to fabricate various microstructures from natural materials^28,29^, and to integrate these materials with microelectronic devices^30^. In addition, excimer laser micromachining has been used as an effective top–down rapid production technique to produce microstructures in natural materials^31^. Bringing together these technologies to bear on natural products such as ECM materials, we created implantable ECM-microelectrodes integrated with insertion stents at high throughput, while preserving the biological nature of these materials. The insertion stents can help minimize damages due to implantation by (a) minimizing the required insertion force and (b) allowing for smaller device footprint^32^.

**Fig. 1:**
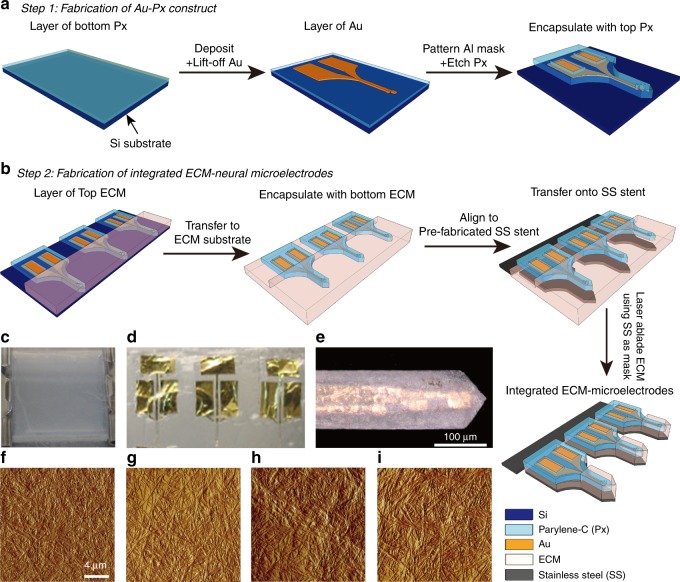
Schematic illustration and images corresponding to steps for fabricating ECM- microelectrodes integrated with insertion stents. **a** Steps for fabricating Au–Px construct, and **b** transfer them onto ECM substrate, integrate with SS stent, and ablate the using excimer laser. Images showing **c** ECM hydrogel (bulk), **d** ECM-microelectrodes, **e** tip and recording sites of an ECM-microelectrode (scale bar: 100 μm); AFM images of ECM substrates: **f** collagen I, **g** collagen I/collagen IV, **h** collagen I/fibronectin, **i** collagen I/laminin

Figure 1a, b schematically illustrates the key processes involved in the fabrication of ECM-microelectrodes integrated with removable insertion stents. The ECM-microelectrodes comprise the functional Au–Parylene C (Px) (pre-ECM) microelectrode with the ECM encapsulation layers, serving as bio-compatible neural interfaces. Four types of ECM-microelectrodes: collagen I microelectrodes, collagen I/collagen IV microelectrodes, collagen I/fibronectin microelectrodes, and collagen I/laminin microelectrodes, each with dimensions (100 μm in width, 30 μm in thickness, and 5 mm in length) comparable to those of commercial silicon M15-microelectrodes (5 mm in length, 80 μm in width at the tip and 300 μm in width at the base, and thickness of 15 μm), were realized using these microfabrication techniques. The collagen I microelectrode is composed of collagen I, while the latter three are composed of ECM composites of collagen I (as structural support) and brain ECM proteins including collagen IV, fibronectin, and laminin. These ECM encapsulations provided adequate mechanical strength required during the fabrication processes. In addition, the long-fibrous morphology of collagen I prepared in this study (Fig. 1f, i) exhibited dimensions (sub-micrometer in diameter and tens of micrometers in length) similar to those of native collagen fibers^33^, thereby simulating a more in vivo-like microenvironment^34^. To aid in insertion, ECM-microelectrodes were physically adhered to insertion stents fabricated from 25-μm-thick stainless steel (SS); as will be described below, this physical adhesion degraded upon post-insertion exposure to wet environments allowing selective withdrawal of the insertion stent. The tensile and bending stiffnesses of these SS-integrated ECM-microelectrodes were calculated to be ~56.3 kN m^−1^ and ~2.20 kN m^−1^, respectively. These are comparable to those of conventional silicon-based microelectrodes, ~151 kN m^−1^ and ~2.13 kN m^−1^ (ref. ^35^), respectively. This enabled insertion of the otherwise flexible ECM-microelectrodes into the desired depth. Testing of the insertion mechanism in vitro using a brain phantom (1% agarose gel) was performed to ensure (i) no premature detachment of the ECM electrode from the insertion stent during the insertion process; (ii) successful detachment and withdrawal of the insertion stent upon completion of insertion; and (iii) preserved functionality of the ECM electrode as assessed by electrical impedance spectroscopy (EIS). For both these in vitro and subsequent in vivo implantations, an insertion speed of approximately 2 mm s^−1^ was used, which resulted in a total insertion time of approximately 1 s. No premature detachment was observed, indicating that the adhesion between the surfaces of the SS stent and dry ECM-microelectrodes was sufficient to withstand the effect of shear forces that arose during implantation. It is likely that this insertion time is much less than the time required for fluid to diffuse from the host tissue to the SS stent–ECM microelectrode interface, reducing the risk of premature separation during insertion. Within 5 min post-insertion, sufficient hydration of the ECM materials was observed, separating the ECM-microelectrodes from the insertion stent. The insertion stent could then be removed, thereby leaving the ECM-microelectrodes implanted. Functionality of the ECM-electrodes implanted using this technique is discussed below.

### Impedance characterization of ECM-microelectrodes

**Fig. 2:**
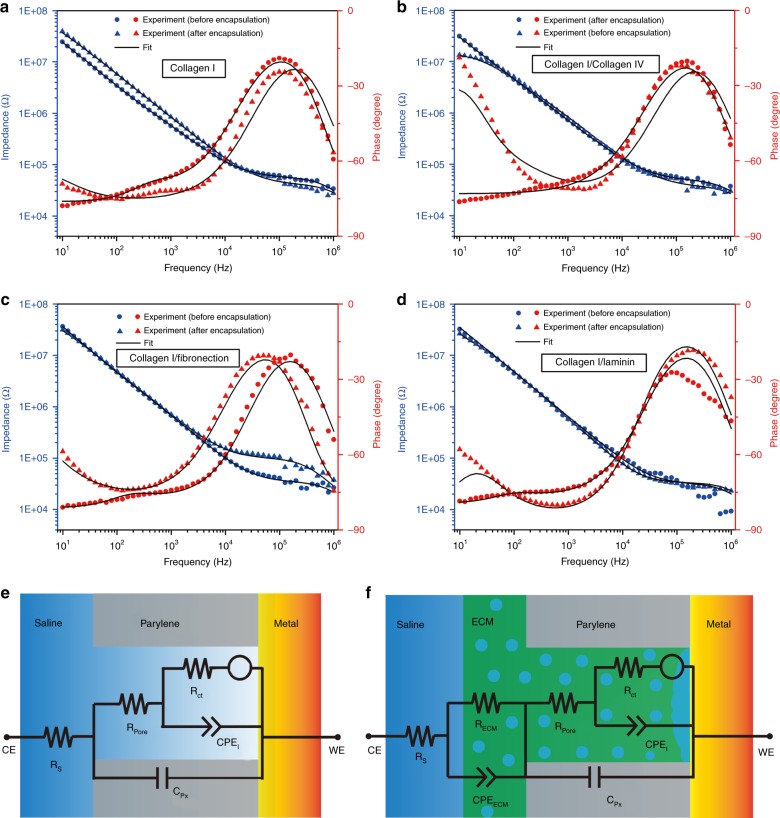
Electrochemical impedance of ECM-microelectrodes. **a**–**d** Bode plot for **a** collagen I microelectrode, **b** collagen I/collagen IV microelectrode, **c** collagen I/fibrinectin microelectrode, and **d** collagen I/laminin microelectrode. Measurement results are shown with symbols and fitting results are shown with solid lines. **e**–**f** Physical representation of the equivalent circuit models for **e** pre-ECM-microelectrodes and **f** ECM-microelectrodes

As the neural recording functionality of the microelectrodes typically relies on their electrochemical impedance characteristics, EIS measurements were performed on ECM-microelectrodes as well as corresponding pre-ECM-microelectrodes in order to understand the influences of ECM encapsulations on the electrochemical impedance characteristics of the neural microelectrodes. For each electrode tested, after ten sequential runs, no degradation in EIS performance was observed. Fig. 2a–d shows magnitude and phase behavior of electrochemical impedances as a function of frequency for the various ECM encapsulations considered in this study. In all cases, the magnitude of impedance was found to decrease monotonically with frequency and its value at the recording frequency of 1 kHz was found to vary between 550 kΩ and 900 kΩ, which are suitable for extracellular recording^36–38^. In comparison to the impedance magnitude, the observed trend of phase angle vs. frequency was more complex. Also, from Fig. 2a–d, it can be seen that the phase behavior of ECM-microelectrodes is noticeably different from its corresponding pre-ECM counterpart.

### Model description

These trends in electrochemical responses were related to the electrical properties of constituent electrode materials by fitting the impedance data to equivalent circuit models shown in Fig. 2e, f. While the overall circuit architecture of these models bears some similarity to that proposed recently by Shi et al.^39^, they differ in their application, both in terms of the phenomena being modeled as well as the design of electrodes and materials employed. The circuit elements in Fig. 2e, f are hereby described in reference to the physical phenomena that they model. The capacitor, *C*_Px_, reflects the dielectric properties of the Px layers and the resistor, *R*_pore_, quantifies the resistance to the motion of ions within the opening in the uppermost Px layer that leads up to the Au–electrolyte interface. A Kovacs circuit was adopted to model the Au–electrolyte interface as a constant phase element, *CPE*_I_, representing the interfacial capacitance, in parallel with a series combination of charge transfer resistance, *R*_ct_, and diffusion-based semi-infinite Warburg element, *W*. The Warburg element, *W*, accounts for impedance arising from ion diffusion, while *R*_ct_ describes the impedance to Faradaic reactions. We model the effects of porous ECM encapsulation through an ad-hoc circuit, comprising a parallel arrangement of constant phase element, *CPE*_ECM_, and a resistor, *R*_ECM_. Here, *CPE*_ECM_ describes the dielectric properties of the ECM encapsulation while *R*_ECM_ represents the resistance to movement of ions through its pores.

### Results from model fit

To obtain the parametric values of these circuit elements (except *R*_ct_), we fit the impedance characteristics of pre-ECM and ECM-microelectrodes, which are presented in Fig. 2a and d, to the circuit models shown in Fig. 2e, f, respectively. This was performed using Echem Analyst, a proprietary software developed by Gamry. We estimated *R*_ct_ via cyclic voltammetry, from the slope of the current vs. overpotential plot (Supplementary Figure S1) using the low field approximation to the Butler–Volmer equation. Thereby *R*_ct_ was determined to be 3.6 MΩ, and was fixed at this value while fitting models. The fitting routine used in this study followed a two-step procedure. First, the measured impedance data of pre-ECM electrodes were fit to the circuit shown in Fig. 2e and the values of corresponding circuit elements were determined. Of these, *C*_Px_, *CPE*_I_, and *R*_s_ were assumed to be largely unaffected by subsequent ECM encapsulation. Next, the impedance data of ECM-microelectrodes were fit to the circuit model shown in Fig. 2f using exactly these values of *C*_Px_, *CPE*_I_, and *R*_s_ to determine the values of remaining circuit elements.

Results obtained from this fitting routine for both pre-ECM and the corresponding ECM-microelectrode samples are illustrated by solid lines in Fig. 2a–d. Also, parametric values corresponding to circuit elements comprising Fig. 2e, f are summarized in Supplementary Table S1 and Table 1. *CPE*_ECM_ and *R*_ECM_ for various ECM encapsulations were found to typically lie in ranges of (1.82–28.7) × 10^−10^ S×*s*^*n*^ (*n*_ECM_ varied in a range of 0.88–1) and 5–21 MΩ, respectively. Overall, the impedance values of ECM-microelectrodes at 1 kHz showed a moderate increase from their corresponding pre-ECM electrodes, as summarized in Table 1.

**Table 1:** Summary of the EIS measurement and fitted parameters of the ECM-microelectrodes

		ECM-microelectrodes			
		Collagen I	Collagen I/Collagen IV	Collagen I/Fibronectin	Collagen I/Laminin
*CPE*_I_ (10^−10^ S×*s*^*n*^)	*Q*_I_	11.8	11.5	7.78	9.07
	*n*_I_	0.83	0.81	0.86	0.85
*R*_ct_ (MΩ)		3.60	3.60	3.60	3.60
*W* (10^−9^ S×*s*^1/2^)		1.92	15.3	3.41	1.56
*C*_Px_ (pF)		4.12	4.14	5.84	5.44
*R*_s_ (kΩ)		1.39	1.90	1.65	1.42
*R*_pore_ (kΩ)		39.5	47.5	91.1	99.6
*CPE*_ECM_ (10^−10^ S×*s*^*n*^)	*Q*_ECM_	1.82	8.55	2.39	28.7
	*n*_ECM_	0.88	1.00	1.00	0.96
*R*_ECM_ (MΩ)		20.5	5.81	6.34	6.38
$|Z|_{{\rm pre} - {\rm ECM}}$ (kΩ)		575	732	674	581
$|Z|_{{\rm post} - {\rm ECM}}$ (kΩ)		852	791	707	583

### ECM-microelectrodes enhanced neuronal viability and neurite outgrowth

**Fig. 3:**
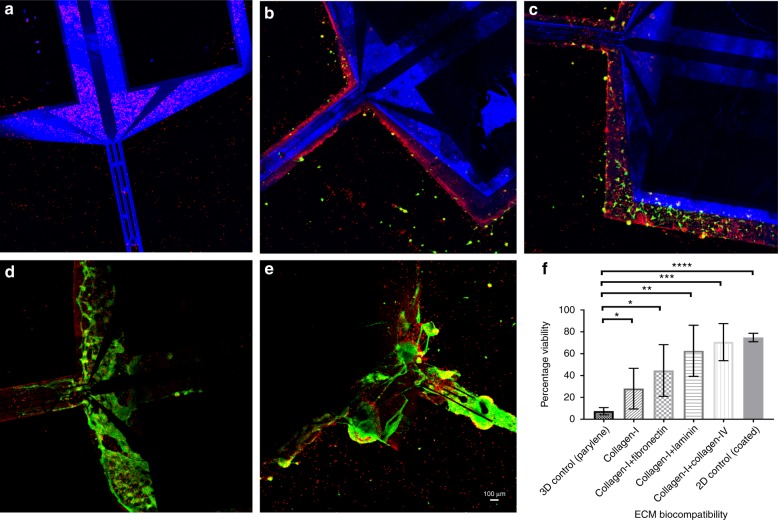
In vitro biocompatibility of ECM-microelectrodes. Live–dead assay of cortical neurons stained with calcein-AM (green) and ethidium homodimer (red) after 7DIV culture on electrodes encapsulated with **a** parylene, **b** collagen I, **c** collagen I/fibronectin, **d** collagen I/laminin, and **e** collagen I/collagen IV. Scale bar = 100 µm. **f** Percentage viability of cells grown over neural electrodes. Unpaired *t*-test was performed on all the groups in comparison with parylene-coated electrodes; *p* < 0.05 (*)

The cytocompatibility of ECM-microelectrodes was assessed based on their ability to support the growth and viability of primary cerebral cortical neurons in vitro. Here, neurons were cultured directly on pre-ECM-microelectrodes (negative control) and ECM-microelectrodes comprising collagen I, collagen I/fibronectin, collagen I/laminin, or collagen I/collagen IV, with comparison to neurons grown in planar culture on polystyrene coated with poly-d-lysine + laminin (positive control). Neuronal viability was poor on pre-ECM-microelectrodes or collagen I microelectrode, but was improved with the addition of fibronectin, laminin, or collagen IV (Fig. 3). In particular, neurons grown on ECM-microelectrodes with additional laminin or collagen IV revealed a markedly increased area of viable cells as compared to pre-ECM-microelectrodes or collagen I microelectrodes. Subsequent calculation of the culture viability ratio revealed that all of the ECM-microelectrodes had significantly higher cell viability as compared to the pre-ECM-microelectrodes group (*p* < 0.05) (Fig. 3f). Out of the experimental ECM-microelectrode groups, a maximum mean culture viability of 70.6% was found for the collagen I/collagen IV microelectrode group, which was comparable to the viability observed for the planar control cultures (74.7%). Further, the skeletal framework of all the electrodes exhibited blue autofluorescence which can be attributed to the presence of parylene^40^.

**Fig. 4:**
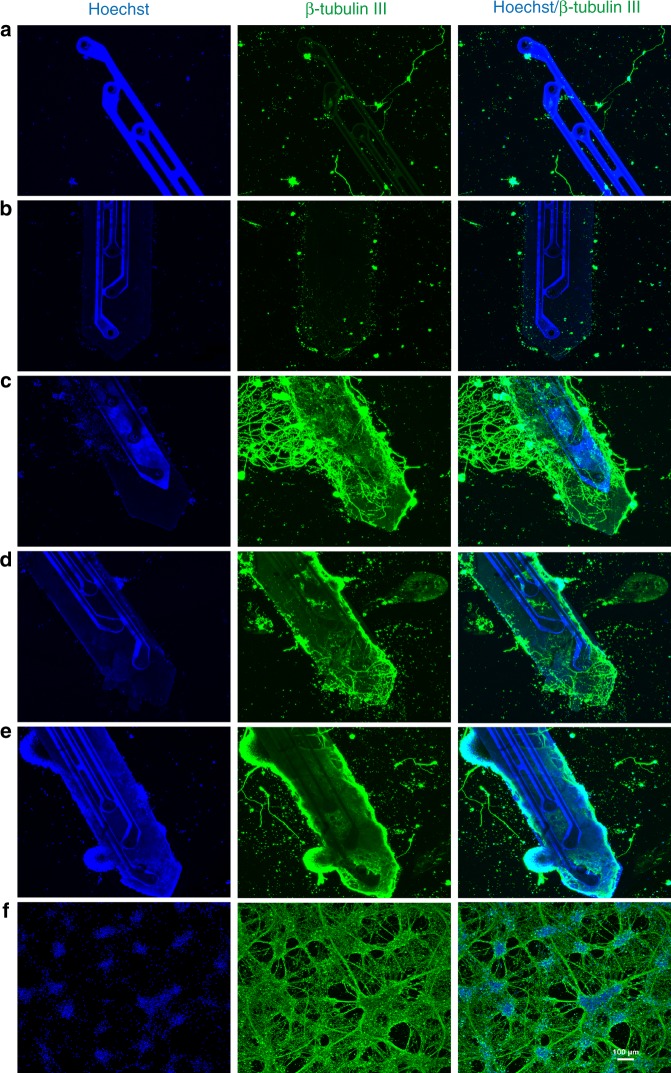
Immunocytochemical analysis of cortical neurons growing on neural electrodes. Confocal fluorescent imaging shows expression of neuronal marker β-tubulin III (green) and nuclei-specific stain Hoeschst (blue) by cortical neurons grown on neural electrodes encapsulated with **a** parylene, **b** collagen I, **c** collagen I/fibronectin, **d** collagen I/laminin, and **e** collagen I/collagen IV in comparison with **f** 2D control group coated with PDL + laminin. Scale bar = 100 µm

In addition, immunocytochemistry was performed using a neuronal cytoskeletal marker (β-tubulin III) to assess neurite outgrowth and network formation on the ECM-microelectrodes. Consistent with the viability assay, we found that the pre-ECM-microelectrode and the collagen I microelectrode groups exhibited minimal cell adherence on the microelectrode surface, as the Px and Au structures were completely visible (Fig. 4a, b). However, ECM-microelectrodes with fibronectin, laminin, or collagen IV were found to have a dense layer of neuronal somata and neurites on the microelectrode surface, as shown by significant expression of β-tubulin III (Fig. 4c, e). Similar neural network growth was observed in two-dimensional (2D) planar cultures, although the network topography was more homogeneous likely due to the uniform geometry in planar culture (Fig. 4f). These findings demonstrate the cytocompatibility of the ECM-microelectrodes and their ability to support the survival and growth of primary cortical neurons with the additional application of fibronectin, laminin, or collagen IV.

### ECM-microelectrodes reduce the foreign body response

**Fig. 5:**
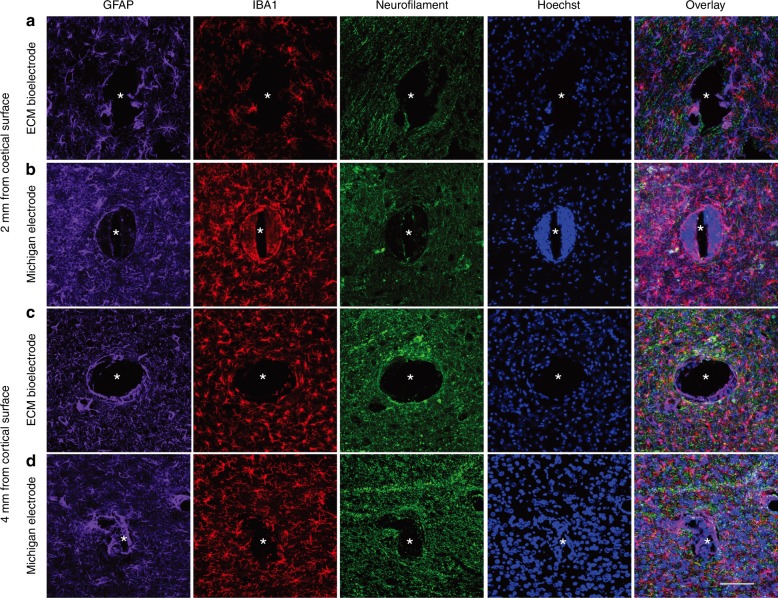
In vivo comparison of ECM-microelectrodes and silicon microelectrodes. **a** Immunohistochemical analysis of ECM-microelectrodes 2 mm below the cortical surface showed minimal glial reactivity and preservation of axonal integrity. **b** Corresponding analyses of silicon microelectrodes (Michigan M15 electrodes) at the same depth exhibits increased astrocytic reactivity and microglial density. **c** At 4 mm below the cortex, ECM-microelectrodes continue to display healthy axons and minimal glial reactivity. **d** Michigan electrodes at this depth yield reduced microglial infiltration but still induce hypertrophy of astrocytic processes. Electrode centers are indicated by asterisks. Scale bar = 50 µm

The neuropathological assessment of the local host cell/tissue responses to implanted collagen I microelectrodes was performed, using a commercially available silicon-based microelectrode as a control. The presence of the stiff insertion stent facilitated precise intracortical implantation of the collagen I microelectrodes. The sub-hundred micrometer size augmented intracortical assimilation of the electrode in rodents. At 1-month post implant into the rat brain, tissue sections orthogonal to the trajectory of the microelectrodes were examined using a panel of immunohistochemical markers and high-resolution confocal microscopy. In particular, we assessed localized reactive astrocytosis, microglial/macrophage recruitment and activation, general cell recruitment and density, and neuronal/axonal loss in the vicinity of the microelectrodes (Fig. 5). An exacerbated neuroinflammatory response to silicon-based control microelectrodes in comparison to collagen I microelectrodes was observed. For example, at more superficial depths (2 mm below the cerebral cortical surface), silicon-based microelectrodes induced a greater astrogliotic response (based on GFAP immunoreactivity and hypertrophy) both at the tissue-microelectrode interface as well as in the general vicinity of the microelectrodes. Here, there was an apparent loss of astrocyte domain organization as there was an increase in (a) astrocyte density and (b) thick hypertrophic somata/processes extending toward and surrounding the silicone microelectrodes. Astrocytes near the collagen I microelectrodes generally presented finer, non-reactive processes and appeared to maintain unique domain organization. Also at superficial depths, exacerbated microgliosis (based on intensity and extent of Iba1 immunoreactivity) was observed around the silicon-based microelectrodes compared to the collagen I microelectrodes. Although most microglia did not appear to be phenotypically reactive (i.e., ameboid) in either group, there was a greater density of Iba1+ cells infiltrating toward the silicon-based microelectrodes relative to the collagen I microelectrodes. Interestingly, these differences in astrocytosis and microgliosis mostly subsided at greater depths, as peri-electrode glial density was relatively similar for each microelectrode by approximately 4 mm below the cortical surface (Fig. 5). In addition to assessing glial changes, we also examined axonal density and integrity, which revealed no differences in neurofilament staining in the vicinity of the microelectrodes for the two groups. However, the most remarkable difference between animals implanted with silicon-based microelectrodes and collagen I microelectrodes was the total cell density near the microelectrodes in general, and directly at the microelectrode–tissue interface in particular. Specifically, we observed a dense layer of host cells lining the silicon-based microelectrodes that appeared consistent with a glial scar. However, the cellular constituents of this dense cell layer only partially labeled for Iba1 and GFAP, suggesting these may predominantly be other infiltrating cell types associated with the glial scar such as endothelial cells and fibroblasts. Of note, this dense cell layer was virtually absent from the interface with the collagen I microelectrodes across all depths examined. These findings demonstrate a differential foreign body response between collagen I microelectrodes and silicon-based microelectrodes at 1-month post-implant, with collagen I microelectrodes eliciting a markedly diminished neuroinflammatory and glial scarring response.

### In vivo neural recording

**Fig. 6:**
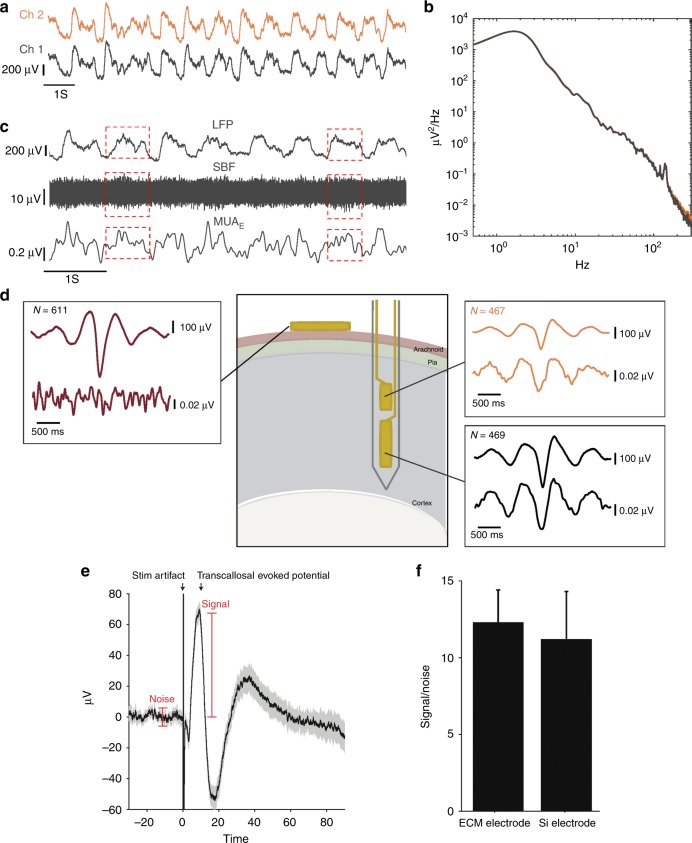
In vivo recording of spontaneous and evoked neural activity with ECM-microelectrodes in the rat cortex. **a** Representative 10-s segments of the local field potential (LFP) recordings from the two channels spaced 190 μm apart. **b** Power spectral density of the neural recordings. **c** Representative 6-s segments of LFP (top), spike-band filtered signal (SBF, middle), and MUA_E_ (multiunit activity envelope, bottom) from Channel 2 (electrode close to the tip). The MUA_E_ was obtained from full-wave rectification and 500 Hz low-pass filtering of the SBF. **d** Schematic representation of the surgical placement of the ECM–intracortical microelectrode and micro-electrocorticography (microECoG) surface electrodes. Side panels show cycle-triggered averages (CTAs) of LFP (top trace) and MUA_E_ (bottom trace) centered on the trough of the down states of the slow (1–2 Hz) LFP oscillation. **e** Example transcallosal evoked potential (EP) recorded by the ECM-microelectrode in response to microstimulation of the opposite brain hemisphere. The signal-to-noise ratio (SNR) of the EP was quantified as indicated. **f** SNR of the EP recorded by the ECM-microelectrode and by a silicon microelectrode (mean ± standard deviation). The difference in mean SNR was not significant (unpaired *t*-test, *p* > 0.05)

To verify the recording functionality of the ECM-microelectrodes, measurements were made using collagen I microelectrodes implanted into the cortex of an anesthetized rat. Fig. 6a and c provides examples of local field potentials (LFP, 0.1–300 Hz) recorded on a collagen I microelectrode. The recordings show slow oscillations, at approximately 1.5 Hz, typical of the anesthetized state. Consistently, the power spectra show a clear physiological peak at 0.5–3 Hz (Fig. 6b). Next, we isolated neuronal action potential activity by filtering the data in the spike-band frequency (SBF, 300–5000 Hz). The root-mean-square of the SBF data, referred to as the multiunit activity envelope (MUA_E_), showed substantial modulation. This result demonstrates that the collagen I microelectrodes could record aggregate neuronal activity intracortically (Fig. 6c). Finally, we performed another experiment in a different pair of anesthetized rats to determine whether the collagen I microelectrode conferred any functional benefit over uncoated microelectrodes. A collagen I microelectrode or a commercial silicon microelectrode (NeuroNexus Technologies) was implanted into the whisker sensory cortex of the two rats. A bipolar stimulating electrode was implanted into the homologous area of the opposite brain hemisphere. Biphasic stimulus pulses (100 µA, 0.2 ms phase^−1^) were delivered and the resulting transcallosal evoked potential (EP) was recorded (Fig. 6e). The signal-to-noise ratio of EP was in the range of 10–15 for both ECM-electrodes and silicon electrodes and did not significantly differ (*t*-test, *p* > 0.05) (Fig. 6f). Thus, the ECM encapsulation did not affect the recording performance of the electrodes acutely. We expect the beneficial effects of the ECM encapsulation will manifest over longer time scales. We are currently working to assess these chronic effects.

## Discussion

The unique biocompatibility of ECM-microelectrodes provides major advantages for neural interfaces over existing devices based on synthetic materials. However, the production of these devices with footprint in micrometer sizes has been a technical challenge due to the incompatibility of the ECM materials with conventional microfabrication methods. The fabrication techniques presented in this work enabled the realization of implantable ECM-microelectrodes with dimensions that are comparable to commercial Si microelectrodes. These techniques allowed batch fabrication of tens of ECM-microelectrodes, with potential for high volume scale-up at a lower cost. Moreover, the same fabrication process can be utilized to produce larger arrays of ECM-microelectrodes with greater number of electrodes. The recording components of these ECM-microelectrodes are effectively encapsulated with thick ECM composites that serve as a biocompatible and electrochemical interface between the microelectrodes and the neural environment.

### Validation of equivalent circuit model

The ECM-microelectrodes possess suitable electrochemical impedance characteristics for neural recordings, which can be analytically described using the equivalent circuit model proposed in this study. From Fig. 2a–d, it can be seen that the proposed circuit models faithfully describe the electrochemical responses of both pre-ECM as well as ECM-microelectrodes over a wide range of frequencies. The fact that this can be achieved by deactivating or activating the ad-hoc circuit shown in Fig. 2f, while retaining identical values of *C*_Px_, *CPE*_I_, and *R*_s_ confirms that the model can capture the underlying physics. Further, the value *C*_Px_ and the impedances due to *CPE*_I_ predicted by the EIS model were also compared to their theoretical estimates. For *C*_Px_, a COMSOL simulation of the pre-ECM-microelectrode in phosphate buffered saline (PBS) (conductivity of 1.4 S m^−1^ (ref. ^41^)) was performed and a value of 1 pF was obtained. This corresponds reasonably well with the model prediction of 4.93 ± 0.95 pF. For *CPE*_I_, the parametric values, *Q*_I_ and *n*_I_, corresponding to various pre-ECM electrodes listed in Table 1 were used to calculate the impedance magnitude, *Z*_CPE-I,EIS_, at $\omega = 1\frac{{{\rm rad}}}{s}$. This value was found to be 1.03 ± 0.02 GΩ. To determine its corresponding theoretical value, *Z*_CPE-I,th_, at $\omega = 1\frac{{{\rm rad}}}{s}$, Eq. 1 was used to calculate the interfacial capacitance, in accordance with the Gouy–Chapman–Stern model^42^.

1 $$Z_{{\rm CPE} - {\rm I,th}}\left( {\omega = 1\frac{{{\rm rad}}}{s}} \right) = \frac{1}{{C_I}} \\ = \frac{{d_{{\rm OHP}}}}{{A{\it{\epsilon }}_0{\it{\epsilon }}_{\rm r}}} + \frac{{L_{\rm D}}}{{A{\it{\epsilon }}_0{\it{\epsilon }}_{\rm r}\cosh \left( {\frac{{zV_{{\rm OHP}}}}{{2V_{\rm t}}}} \right)}}.$$

Here, ${\it{\epsilon }}_0$ is the permittivity of free space, ${\it{\epsilon }}_{\rm r}$ is the relative permittivity of the double layer, *d*_OHP_ quantifies the thickness of the outer Helmholtz plane (OHP), *z* is the ionic charge in the solution, and *A* is the exposed area of the recording site. Also, *V*_OHP_ is the potential at OHP, while *V*_t_ and *L*_d_, represent thermal voltage and Debye length, respectively. These were determined through Eqs. 2–4 (ref. ^42^):

2 $$-12pt V_{{\rm OHP}} = V_0 - \left( {\sqrt {\frac{{8qV_{\rm t}n_0}}{{{\it{\epsilon }}_0{\it{\epsilon }}_{\rm r}}}} } \right)\left( {d_{{\rm OHP}}{\rm sinh}\left( {\frac{{zV_{{\rm OHP}}}}{{2V_{\rm t}}}} \right)} \right),$$

3 $$V_{\rm t} = \frac{{kT}}{q},$$

4 $$L_{\rm D} = \sqrt {\frac{{{\it{\epsilon }}_0{\it{\epsilon }}_{\rm r}V_{\rm t}}}{{2n_0z^2q}}}.$$

Here, *V*_0_ is the applied electrode potential, *n*_0_ is the concentration of ions in the bulk of the solution, *k* is the Boltzmann constant, and *q* is the elementary charge. The values corresponding to remaining constants that comprise Eqs. 1–4 are listed in Table S2 in the supplementary information.

In using Eqs. 1–4, PBS was assumed to be a symmetric 1:1 electrolyte comprised of NaCl (137 mM) and KCl (2.7 mM). Although this approximation is inexact, due to the presence of phosphate buffer, it provides a resonably accurate estimate of *Z*_CPE-I,th_, given that the ionic strength of the electrolyte can be attributed mostly to NaCl. Under this assumption, by using Eqs. 1–4, the value of *Z*_CPE-I,th_ at $\omega = 1\frac{{{\rm rad}}}{s}$ was calculated to be 0.99 GΩ, which is almost exactly equal to that predicted by EIS (1 GΩ).

Therefore, the equivalent circuit models presented in this study provide a good numerical fit as well as a physiological understanding of the recorded electrochemical impedance responses of ECM-microelectrodes. This can facilitate better design and integration of ECM materials as bio-compatible encapsulations for several applications, such as, neural prosthetic devices and therapeutic applications, which typically record slow rhythm field potentials (<200 Hz) and action potentials (0.1–7 kHz)^1^ as well as biosensors operating in the radio frequency spectrum (~MHz)^43–46^.

### Enhancing biocompatibility with ECM-microelectrodes

ECM proteins such as collagen, fibronectin, and laminin improve neuron-to-electrode surface attachment^47^. However, collagen IV in particular has been shown to promote neurogenesis and inhibit glial proliferation in rat cortical progenitor cell cultures derived from E16 fetuses^48^. In our study, ECM-microelectrodes developed using collagen I composites with laminin, fibronectin, and collagen IV exhibited enhanced neuronal viability and superior cell adhesion and network formation in comparison to groups with collagen I microelectrodes and pre-ECM-microelectrodes. We also observed that collagen I/collagen IV microelectrodes performed best in terms of neuronal viability (Fig. 3f), cellular density on electrode surface, and expression of the axonal marker β-tubulin (Fig. 4e), thereby providing enhanced biocompatibility with neuronal environment compared with collagen I microelectrodes and pre-ECM-microelectrodes. In addition to the ECM proteins utilized in the present study, various ECM molecules exhibit functionalities in promoting neuronal cells survival and regulating various aspect of neuronal physiology. Although in the present study, only major ECM proteins are used to demonstrate the device fabrication and biocompatibility, integration of different biological and topological cues are expected to provide additional functionality for neural interfaces.

### Tissue responses to ECM-microelectrodes

The in vivo histological results demonstrated that collagen I microelectrodes elicited attenuated neuroinflammation in the surrounding parenchyma compared with commercial silicon-based microelectrodes, indicating that these collagen I microelectrodes may cause significantly less tissue damage via infiltrating cells over time. Chronic microgliosis and astrogliosis can contribute to neuronal cell damage, therefore it is remarkable that these processes appeared to be limited in rodents up to 1 month following collagen I microelectrode implantation. Furthermore, reactive astrocytes can accumulate and impede the efficacy of implanted electrodes^49,50^. The observed reduction of gliotic pathways illustrates that the collagen I microelectrodes utilized in this study may possess advantageous traits that grant enhanced biocompatibility while minimizing cellular damage. Additionally, axonal processes did not appear to be structurally perturbed by the collagen I microelectrodes, suggesting that these electrodes may be implanted with minimal damage to functional neural networks. This phenomenon further indicates that collagen I microelectrodes can be assimilated along subcortical white matter tracts with minimal disruption of the surrounding neuronal processes.

### Recording capability of ECM-microelectrodes

To confirm that the modulated SBF activity recorded on the collagen I microelectrodes was generated by firing of local neuronal microcircuits, we performed an additional analysis on the MUA_E_ and compared the results to those from a control microECoG electrode placed above the brain surface, as shown in Fig. 6d. The MUA_E_ provides an instantaneous measure of the aggregate activity of the neuronal population proximal to the electrode. Upward deflections represent increased activity^51^, which in this recording from the collagen I depth microelectrodes, correlated with the up states of the slow LFP oscillation (highlighted by dashed boxes in Fig. 6c). The cycle-triggered averages (CTAs) of LFP quantify the average slow oscillation profiles, which were similar between the collagen I microelectrodes and on the microECoG, as expected given the global nature of the field potential (Fig. 6d). The MUA_E_ CTAs quantify how well the local neuronal activity was correlated with the LFP oscillations. At the brain surface, the MUA_E_ CTA was relatively flat, indicating that local MUA was not modulated by the slow rhythm or, more likely, that MUA was not detectable at this site. In contrast, the MUA_E_ CTAs of the intracortical sites show a modulation that mirrors the LFP CTAs, indicating that up/down states of each slow oscillation cycle are associated with more/less neuronal activity, respectively, as seen in Fig. 6d. Furthermore, the MUA_E_ CTA appears to have higher amplitude at the deeper intracortical site, likely reflecting the larger aggregate signal generated by infragranular pyramidal neurons. The in vivo recordings therefore confirmed the ability of collagen I microelectrodes to record both local as well as global rhythms in neuronal activities. Although only collagen I microelectrodes are used here to demonstrate the neural recording capability of the ECM-microelectrodes, due to the fact that collagen I/collagen IV microelectrodes, collagen I/laminin microelectrodes, and collagen I/fibronectin microelectrodes present similar EIS characteristics, they are expected to show similar performance for neural recordings compared with the collagen I microelectrodes. In addition to the above-mentioned ECM-microelectrodes, the microelectromechanical systems (MEMS) technologies proposed in this study will make possible a variety of ECM-microelectrodes with favorable biological properties, suitable electrochemical characteristics, as well as proper geometric and mechanical features for a broad range of neural interfacing applications.

## Materials and methods

### Preparation of ECM composite films

Four different ECM films (collagen I film, collagen I/collagen IV composite film, collagen I/fibronectin composite film, and collagen I/laminin composite film) were prepared as follows: Type I rat tail collagen in a 3 mg mL^−1^ solution (Corning®, Corning, NY) was combined with 10× PBS and 0.1 M NaOH at a ratio of 13:2:1 by volume, to form a base solution. To form a Type IV/Type I collagen ECM solution, Type IV mouse collagen in a 1 mg mL^−1^ solution (Corning®, Corning, NY) was added to this base solution such that the total collagen content comprised 92% Type I collagen and 8% Type IV collagen by weight. Similarly, to form a fibronectin/Type I collagen ECM solution, fibronectin powder from rat plasma (Sigma Aldrich®, St. Louis, MO) was added to the base solution such that the total protein content comprised 92% Type I collagen and 8% fibronectin by weight. Finally, to form a laminin/Type I collagen ECM solution, 6 mg mL^−1^ laminin (Trevigen, Gaithersburg, MD) was added to the base solution such that the total protein content comprised 92% Type I collagen/8% laminin by weight. In addition to these three ECM composite solutions, the base solution was used directly as a collagen I ECM solution. Each ECM solution was gently mixed until small fragments started to form. The mixed solutions were then cast into an acrylic mold, followed by polymerization at 37 °C and 96% humidity for 24 h to form ECM hydrogels with fibril structures. These ECM hydrogels were then dried on glass slides in air at 37 °C for 24 h. The ECM films were rinsed with DI water multiple times until fully transparent films were observed. After rinsing, the films were air dried, forming uniform dried ECM films. The thickness of each film was approximately 10 μm.

### Fabrication of pre-ECM microelectrodes

Briefly, a bare silicon wafer was first coated with Px film (5 μm) using an SCS PDS 2010 Parylene coater. The electrode and conductive traces were then lithographically defined on this Px layer, as shown in Fig. 1a. Subsequently, a thin film (100 nm) of Au was deposited by e-beam evaporation and a lift-off process was performed to realize conductive Au traces. To encapsulate the device, a second layer of Px (5 μm) was deposited using the previously mentioned process. To interface with neurons and record their activities, both ends of the conductive Au traces, which we refer as pads hereafter, needed to be exposed. To facilitate this, first, a window type pattern was lithographically defined and registered to the Au layer. A thin film (100 nm) of Al was then deposited using e-beam evaporation and lifted off thereafter to remove Al selectively from the pads. Using the Al pattern as an etching mask, RIE was performed using O_2_ gas to (a) define the electrode footprint and (b) provide access to the pads for interfacing and recording signals. The Al mask was then removed via wet etching. The pre-ECM electrodes used in this study have either two or three recording sites. While the former, which possessed a smaller footprint, was used for in vivo neural recording, the latter was employed for neuronal cell studies. Pre-ECM electrodes with two recording sites had corresponding site area of 1000 μm^2^ (for the site further from tip of the shank) and 2000 μm^2^ (for the site closer to the tip of the shank), respectively. Also, using the same fabrication procedure, microECoG surface electrodes with recording site areas of 2500 μm^2^ were fabricated as controls, to facilitate comparisons between intracortical and intracranial recordings.

### Fabrication of ECM microelectrodes

The distal regions of pre-ECM depth electrodes (which ultimately will be in contact with cortical tissue) were transferred onto ECM films, as shown in Fig. 1b. Following a brief immersion in DI water, these Au–Px–ECM constructs were detached from the underlying Si substrate. The exposed backsides of these devices were then coated with another ECM hydrogel and air dried for 12 h. Thus, complete encapsulation of the pre-ECM electrodes was achieved. Next, to aid the insertion process, an SS stent was laser micromachined with dimensions corresponding to the electrode footprint. The ECM-encapsulated Au–Px constructs were then hydrated and carefully placed onto this pre-patterned steel stent to ensure registration of their respective patterns. Finally, using this stent as a template, the underlying ECM films was patterned via excimer laser micro-machining to realize insertion-stent-integrated ECM-microelectrodes.

### Electrochemical impedance measurements

EIS was performed in 1× PBS over a frequency range from 10 Hz to 1 MHz with a potential amplitude of 20 mV. The EIS was performed using a Gamry Reference 600^TM^ potentiostat (Gamry Instruments, Warminster, PA, United States) in a three-electrode setup where a platinum wire served as the counter electrode and Ag/AgCl electrode as the reference electrodes. Measurements were obtained using ECM microelectrodes having two recording sites. Both sites were found to exhibit similar trends in their impedance magnitude and phase angle response, although the absolute values of these parameters were quite different. This can be mostly attributed to the differences in the areas of the recording sites. Representative results presented in this study were obtained from the larger recording site (area of 2000 μm^2^) located closer to the tip of the electrode shank.

### Charge transfer resistance

Under low-field approximation, the Butler–Volmer equation reduces to Ohm’s law, which yields^52^

$$J = \frac{{J_0zF\eta }}{{RT}},$$

where *J* is the current density under applied overpotential, *η*; *J*_0_ is the equilibrium exchange current density; *z* is the number of electrons involved in the redox reaction; *F* is Faraday’s constant; *R* is the gas constant; and *T* is the temperature. Under this approximation, the charge transfer resistance, *R*_ct_, can be determined by the slope of the current vs. overpotential plot, which gives

$$R_{{\rm ct}} = \frac{{RT}}{{J_0zF}}.$$

### Simulation of *C*_Px_

The value corresponding to *C*_Px_ was obtained from a finite element model of the pre-ECM-microelectrode in PBS solution, where the Au electrode (20 μm in width, 5 mm in length, and 100 nm in thickness; electrical conductivity, *ρ*_Au_, of 0.022 μΩ m) and the 1× PBS solution (*ρ*_PBS_ = 1.4 S m^−1^) occupied either sides of the Px insulation layer (80 μm in width, 5 mm in length, and 5 μm in thickness; dielectric constant, *ε*_r_, of 3.10). The electrostatics model under the AC/DC module of COMSOL Multiphysics Modeling Software (COMSOL Inc., Burlington, MA, United States) was utilized to perform the simulations. The Au electrode was held at a potential of 20 mV while the PBS domain was grounded. A relatively large PBS solution domain was chosen to capture the contributions to *C*_Px_, from fringing electric fields.

### Cell viability assay

Cell viability was assessed following plating on the pre-ECM-microelectrodes and the ECM-microelectrodes (*n* = 5–7 per group) or on planar control substrates (*n* = 3) at 7 DIV as per standard protocols^53,54^. Briefly, neuronal cultures were rinsed with Dulbecco’s phosphate-buffered saline (DPBS), incubated with ethidium homodimer-1 (EthD-1; 4 μM; Life technologies), and calcein AM (2 μM; Sigma) at 37 °C for 30 min, and then rinsed three times with DPBS. Cell viability ratio was calculated by measuring the fluorescent intensity of live cells (green) and that of the dead cells (red).

### Immunocytochemistry

Neuronal adhesion, growth, and network formation were qualitatively assessed via immunocytochemistry for cultures grown on the various ECM-microelectrodes vs. the 2D control cultures (*n* = 1 for each group). At 7 DIV, cultures were fixed in 4% formaldehyde for 35 min, rinsed in PBS, and permeabilized using 0.3% Triton X100 plus 4% horse serum for 60 min. Subsequently, mouse anti β-tubulin III primary antibody (Sigma-Aldrich T8578, 1:500) was added (in PBS + 4% serum) and incubated at 4 °C for 12 h. This antibody binds to β-tubulin III protein, which is a microtubule element expressed primarily in neuronal somata and neurites. After rinsing the primary antibody, an appropriate fluorescent secondary antibody (donkey anti mouse IgG - Alexa-488 at 1:500 in PBS + 4% horse serum) was added at 18–24 °C for 2 h. The secondary antibody solution was then removed, and the cells were incubated with 30 nM Hoechst in PBS for 10 min, followed by repeated rinses with PBS. The cells were stored in PBS at 4 °C before imaging.

### Tissue harvest and immunohistochemistry

At 1-month post-implant, the animals were anesthetized and transcardially perfused with heparinized saline followed by 10% formalin. The brains were removed, post-fixed for 24 h, and prepared for cryosectioning. Brains were put into 30% sucrose until saturated and frozen whole in isopentane and dry ice slurry. Frozen brains were mounted in a cryostat and faced at a plane orthogonal to the microelectrode tract and then sectioned at 35 µm thick. For immunohistochemistry, sections were air-dried for 30 min and rehydrated in PBS three times for 5 min each. Next, sections were blocked with 4% normal horse serum in 0.1% Triton X/PBS for 30–45 min. Primary antibodies were applied to the sections in 4% normal horse serum/Optimax buffer at 4 °C overnight. Sections were triple-labeled using the following primary antibodies: (1) glial-fibrillary acidic protein (GFAP), an intermediate filament protein expressed in all astrocytes that is upregulated in hypertrophic/reactive astrocytes (rabbit anti-GFAP, 1:500, Millipore Ab5804); (2) neurofilament, a cytoskeletal constituent expressed in neurons and enriched in axons (mouse anti-SMI31, 1:1000, Millipore NE1022); and (3) ionized calcium-binding adapter molecule 1 (Iba1), a marker for microglia/macrophages allowing for assessment of morphological changes associated with activation (goat anti-IBA1, Abcam, Ab5076). The next day, sections were rinsed with PBS three times for 5 min each. Alexa Fluor conjugated secondary antibodies (donkey anti-mouse 488, A21202; donkey anti-goat 568, A11057; donkey anti-rabbit 647, A31573; 1:1000) were applied in 4% normal horse serum/PBS for 2 h at room temperature. Sections were counterstained with DNA-specific fluorescent Hoechst 33342 for 5 min and then rinsed with PBS. After immunostaining, slides were coverslipped with Fluoromount-G mounting media.

### Statistical analyses

Outlier tests were performed for all quantitative data. Data from the viability assay were analyzed using an unpaired *t*-test with Welch correction for each group against the pre-ECM group. For all statistical tests, *p* < 0.05 was required for significance. Data are presented as mean ± standard deviation.

### Neural recording

Three Sprague–Dawley rats were used for the in vivo recording experiments. Each rat was anesthetized with an intraperitoneal injection of ketamine (60 mg kg^−1^) and dexmedetomidine (0.25 mg kg^−1^) and placed in a stereotaxic frame. A craniotomy was performed to expose the whisker sensory (i.e., barrel) cortex of the right hemisphere and the dura was removed. A skull screw was placed in the left parietal bone to serve as the reference electrode for the recordings. A collagen I microelectrode or a silicon microelectrode was attached to a micromanipulator and the tip of the array was implanted to a depth of 2 mm below the pia. In two rats, a second craniotomy exposed the barrel cortex in the opposite hemisphere and a concentric bipolar stimulating electrode was implanted at the same depth. Immediately after implantation, neural recordings were acquired with a commercial electrophysiology system (Tucker-Davis Technologies). Single pulse electrical stimuli were delivered at a rate of 0.1 Hz in the EP experiments. Finally, in one rat, a microECoG was placed over the exposed cortical surface and used to record cortical surface potentials. These procedures were approved by the Institutional Animal Care and Use Committee of the University of Pennsylvania.

## Electronic supplementary material

SUPPLEMENTAL MATERIAL
