# Supplementary material for: Microfabricated intracortical extracellular matrix-microelectrodes for improving neural interfaces
Source: Microsyst Nanoeng. 2018 Sep 24;4:30. doi: 10.1038/s41378-018-0030-5 (PMC6220172; doi:10.1038/s41378-018-0030-5)
Supplement: Supplementary file 1 — SUPPLEMENTAL MATERIAL [file 41378_2018_30_MOESM1_ESM.docx]

Microfabricated Intracortical Extracellular Matrix-Microelectrodes for Improving Neural Interfaces

Wen Shen^1^, Suradip Das^2^, Flavia Vitale^3^, Andrew Richardson^4^, Akshay Ananthakrishnan^5^, Laura A. Struzyna^6^, Daniel P. Brown^4^, Naixin Song^7^, Murari Ramkumar^8^, Timothy Lucas^4^, D. Kacy Cullen^4^, Brian Litt^3^, Mark G. Allen^1*^

^1^ Krishna P. Singh Center for Nanotechnology, University of Pennsylvania, Philadelphia, PA 19104, USA
^2^ Department of Neurosurgery, Perelman School of Medicine, University of Pennsylvania, Philadelphia, PA 19104, USA

^3^ Department of Neurology, Perelman School of Medicine, University of Pennsylvania, Philadelphia, PA 19104, USA

^4^ Department of Neurosurgery, Perelman School of Medicine, University of Pennsylvania, Philadelphia, PA 19104, USA

^5^ Department of Mechanical Engineering and Applied Mechanics, School of Engineering and Applied Science, University of Pennsylvania, Philadelphia, PA 19104, USA

^6^ Department of Bioengineering, School of Engineering and Applied Science, University of Pennsylvania, Philadelphia, PA 19104, USA

^7^ Department of Electrical and Systems Engineering, School of Engineering and Applied Science, University of Pennsylvania, Philadelphia, PA 19104, USA

^8^ Department of Materials Science and Engineering, School of Engineering and Applied Science, University of Pennsylvania, Philadelphia, PA 19104, USA

^*^ Correspondence: Professor Mark G. Allen, Krishna P. Singh Center for Nanotechnology, University of Pennsylvania, Philadelphia, PA 19104, USA

E-mail: [mallen@seas.upenn.edu](mailto:mallen@seas.upenn.edu)

**Supplementary Information**

**Cyclic Voltammetry**

Cyclic voltammetry (CV) was performed in a three-electrode configuration as described in the above section. A potential swept between -0.9 V to 0.5 V with respect to the open circuit potential (OCP) was applied on the working electrode at a scan rate of 50 mV/s.

**Neuronal Cell Culture**

All procedures involving animals were approved by the Institutional Animal Care and Use Committees at the University of Pennsylvania and The Michael J. Crescenz Veterans Affairs Medical Center and were carried out in accordance with Public Health Service (PHS) Policy on Humane Care and Use of Laboratory Animals (2015).

Cerebral cortical neurons were isolated from fetuses (embryonic day 18) of Sprague-Dawley rats (Charles River, Wilmington, MA) as described previously [37]–[39]. Briefly, timed-pregnant rats were euthanized through exposure to carbon dioxide followed by decapitation. The uterus was extracted through Caesarian section and each fetus was removed from the amniotic sac and put in cold Leibovitz-15 media. The cerebrum was isolated and the cortices were dissociated in pre-warmed trypsin (0.25%) + EDTA (1 mM) for 12 minutes at 37°C. The trypsin-EDTA was then removed and the tissue was triturated in HBSS containing DNase I (0.15 mg/mL). The cells were centrifuged at 1000 rpm for 3 minutes and resuspended at 1.3x10^6^ cells/mL in Neurobasal medium + 2% B27 + 0.4 mM L-glutamine + 0.1% PenStrep (Cortical Neuron Media).

Microfabricated neural microelectrodes (pre-ECM-microelectrodes, n=8; collagen I microelectrodes, n=8; collagen I/fibronectin microelectrodes, n=6; collagen I/laminin microelectrodes, n=8; collagen I/collagen IV microelectrodes, n=7) were fixed to each well of a 12-well plate using silicon adhesive and sterilized by UV exposure for 30 minutes. For seeding cortical neurons, 50 μL of cell solution was precisely added on the surface of each microelectrode. The cultures were placed in a humidified tissue culture incubator (37°C and 5% CO_2_) for 2 hours to allow cells to attach, after which, 2 mL of Cortical Neuron Media was added to each well. For the 2D control groups (n=4), wells were coated with Poly-D-Lysine (20ug/mL) overnight followed by Laminin (20 ug/mL) for 2 hours, then 50 μL of cortical cell solution was seeded in each well followed by 2 mL of Cortical Neuron Media. Fresh, pre-warmed Cortical Neuron Media was used to replace the culture media every 2-3 days *in vitro* (DIV).

**Surgical Implantation of Microelectrodes**

Male Sprague-Dawley rats weighing 325-350g were used for this experiment. Rats were anesthetized with isoflurane, the head was shaved, and the animals were mounted in a stereotactic frame. The scalp was cleaned with betadine, bupivacaine was injected along the incision line, and a midline incision was made to expose the Bregma landmark. A small craniectomy was made in the anterior part of the skull at the following coordinates in relation to Bregma: AP: -3.0 mm, ML: ±5.8 mm, 38 degrees off of vertical. A small mark was made on the dura using tissue dye. The dura was opened and the “dummy” (non-functional) microelectrode (collagen I microelectrode, 100 μm in width, a total of 30 μm in thickness, and 5 mm in length n=3; silicon microlectrode, 100 μm in width, 15 μm in thickness, and 5 mm in length, n=3) lowered 5 mm into the brain. Once the microelectrode was implanted, the microelectrode holder was removed. The scalp was sutured closed and animals returned to the colony. Meloxicam was provided for analgesia.

**Microscopy and Data Acquisition**

Cell viability, immunocytochemistry, and immunohistochemistry analyses of the cells/tissue were performed using a Nikon A1RSI Laser Scanning Confocal microscope with image analysis performed using Nikon Elements BR 4.10.01. Experienced researchers who were blinded to experimental group performed all analyses. For *in vitro* assays, multiple z-stacks were digitally captured and analyzed to assess cell viability, neuronal adhesion, and neurite outgrowth on the ECM-microelectrodes with various protein contents. In all cases, 2D planar cultures grown on Poly-D-Lysine + Laminin coated wells served as positive controls. For the viability assay, the microelectrode surface area was marked as the region of interest (ROI) for intensity measurements. The culture viability ratio was calculated on the basis of fluorescent intensity as per the following formula:

$$Percentage viability= \frac{(Intensity\text{ }\text{Green})}{(Intensity\text{ }\text{Green }\text{+ }Intensity \text{Red})}\times100$$

For the immunocytochemistry assay, the area of neuronal adhesion, neuronal morphology, and extent of neurite outgrowth and network formation were qualitatively assessed across groups. For immunohistochemistry of brain tissue following microelectrode implant, tissue sections (orthogonal to microelectrode implantation trajectory) underwent a detailed neuropathological analysis of astrocyte reactivity (e.g., GFAP intensity, hypertrophy, density at microelectrode interface), axonal changes (e.g., integrity and density in microelectrode vicinity), microglial/macrophage recruitment (e.g., density and amoeboid/activated morphology), and general cell density changes. This analysis was performed as a function of depth from the cortical surface (1 - 6 mm), with focus on tissue directly at the microelectrode interface and the immediate vicinity.


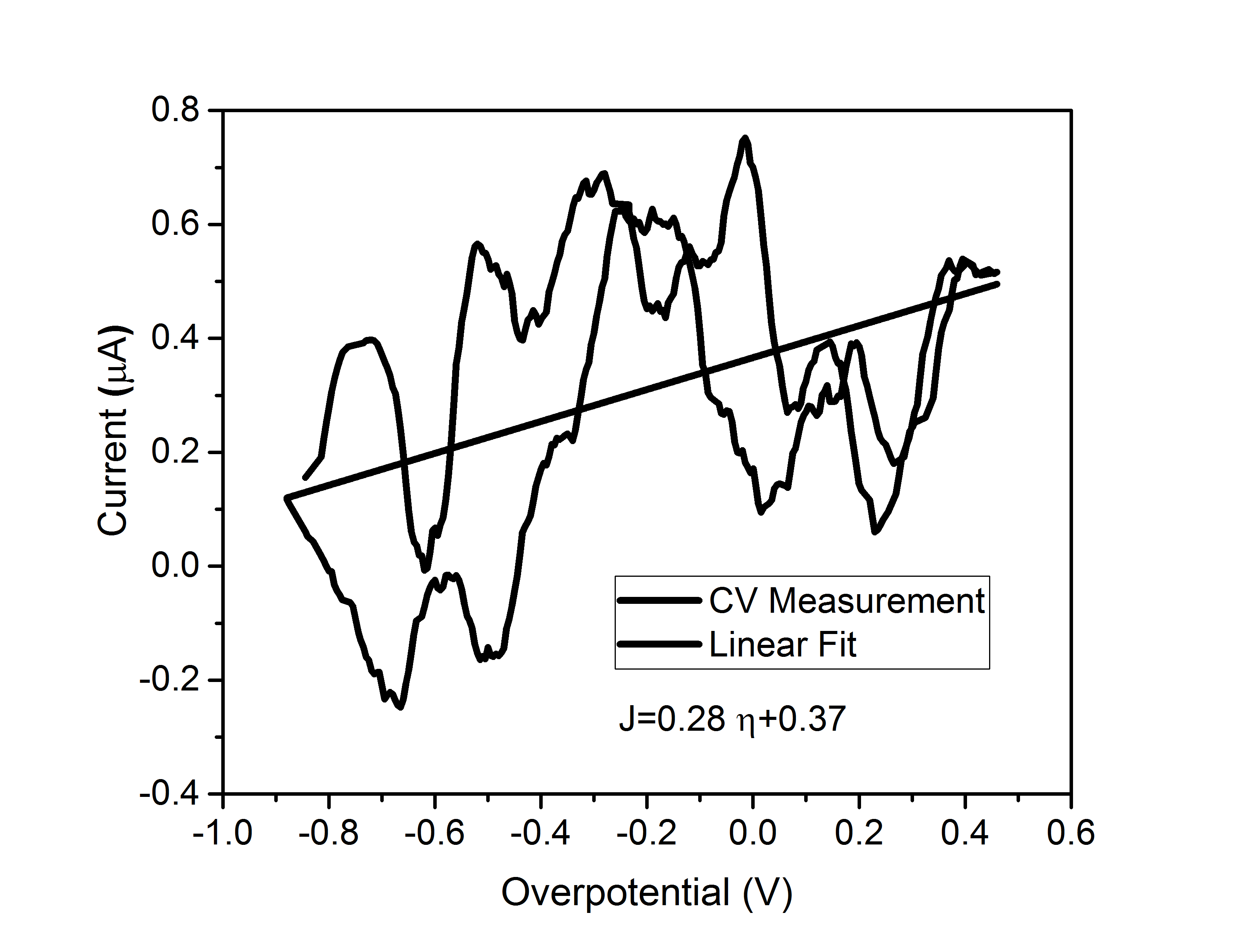


Figure S1. Current density versus applied electrode overpotential. *R_ct_* is estimated as the inverse of the slope.

Table S1. Representative EIS measurement and fitted parameters of pre-ECM and post-ECM microelectrodes.

|  |  | Collagen I | | Collagen I/Collagen IV | | Collagen I/ Fibronectin | | Collagen I/Laminin | |
| --- | --- | --- | --- | --- | --- | --- | --- | --- | --- |
|  |  | pre-ECM | post-ECM | pre-ECM | post-ECM | pre-ECM | post-ECM | pre-ECM | post-ECM |
| ${CPE}_{I}$  (10^-10^ S×s^n^) | $Q_{I}$ | 11.8 | 11.8 | 11.5 | 11.5 | 7.79 | 7.78 | 9.07 | 9.07 |
|  | $n_{I}$ | 0.83 | 0.83 | 0.81 | 0.81 | 0.86 | 0.86 | 0.85 | 0.85 |
| *R_ct_* (MΩ) | | 3.60 | 3.60 | 3.60 | 3.60 | 3.60 | 3.60 | 3.60 | 3.60 |
| $W$ (10^-9^ S×s^1/2^) | | 2.70 | 1.92 | 0.20 | 15.3 | 1.51 | 1.56 | 7.87 | 8.93 |
| $C_{Px}$ (pF) | | 4.12 | 4.12 | 4.14 | 4.14 | 5.44 | 5.44 | 6.00 | 6.00 |
| $R_{s}$ (kΩ) | | 1.39 | 1.39 | 1.90 | 1.90 | 1.42 | 1.42 | 1.00 | 1.00 |
| *R_pore_* (kΩ) | | 56.3 | 39.5 | 47.5 | 47.5 | 34.6 | 99.6 | 30.0 | 21.13 |
| ${CPE}_{ECM}$  (10^-10^ S×s^n^) | $Q_{ECM}$ | / | 1.82 | / | 8.55 | / | 2.39 | / | 28.7 |
|  | $n_{ECM}$ | / | 0.88 | / | 1.00 | / | 1.00 | / | 0.96 |
| $R_{ECM}$ (MΩ) | | / | 20.5 | / | 5.81 | / | 6.34 | / | 6.38 |
| $\vert Z\vert$ (kΩ) at 1 kHz | | 575 | 852 | 732 | 791 | 674 | 707 | 581 | 583 |

**Table S2. Parameters used to calculate the interfacial capacitance.**

| Parameter | Symbol | Value |
| --- | --- | --- |
| Thickness of the outer Helmholtz plane of physiological saline at 25 ºC | $d_{OHP}$ | 5 Å [52] |
| Permittivity of free space | $\epsilon_{0}$ | 8.85 × 10^-12^ F/m |
| Relative permittivity of the double layer at 25 ºC | $\epsilon_{r}$ | 78 [52] |
| Exposed area of the recording site | $A$ | 1890 × 10^-12^ m^2^ |
| Ionic charge in the solution | $z$ | 1 |
| Concentration of ions in the bulk of the solution | $n_{0}$ | 8.413 × 10^25^ ions/m^3^ |
| Applied electrode potential | $V_{o}$ | 20 mV |
| Thermal voltage | $V_{t}$ | 0.0259 V |
| Elementary charge | $q$ | 1.602 × 10^-19^ C |
